# Does conventional laser photocoagulation still have a place in the treatment of diabetic macular edema?

**DOI:** 10.22336/rjo.2021.27

**Published:** 2021

**Authors:** Belma Kayhan, Engin Burumcek

**Affiliations:** * Department of Ophthalmology, University of Health Sciences, Sultan 2. Abdulhamid Han Training and Research Hospital, Istanbul, Turkey; ** Department of Ophthalmology, Osmanoglu Hospital, Istanbul, Turkey

**Keywords:** laser photocoagulation, diabetic macular edema, focal laser, grid laser

## Abstract

**Objective:** The study aimed to investigate the long-term efficacy of conventional laser photocoagulation in the treatment of diabetic macular edema.

**Methods:** In this retrospective study, the records of patients presented with diabetic macular edema (DME) and non-proliferative diabetic retinopathy were reviewed. DME defined as clinically significant macular edema was treated by using argon green or yellow dye laser with focal, grid, and modified grid techniques according to Early Treatment Diabetic Retinopathy Study parameters. Best-corrected visual acuity (BCVA) was measured. BCVA change after the treatment and its relationship with other factors were evaluated.

**Results:** The study included 133 eyes of 81 patients. The mean follow-up was 28.26 months. BCVA demonstrated the increase of 2 lines or more in 20.7% of the eyes, stabilization within 2 lines in 60.7% of the eyes, and loss of 2 lines or more in 18.3% of the eyes. The eyes with baseline BCVA lower than or equal to 0.50 showed a statistically significant increase (p=0.001) whereas the eyes with baseline BCVA of more than 0.50 did not show a statistically significant change (p=0.070) after laser photocoagulation treatment.

**Conclusions:** Conventional laser photocoagulation is an effective treatment in diabetic macular edema including center-involved type and stabilizes visual acuity in the majority of the patients. Improvement in BCVA is significant in the group with lower baseline BCVA.

**Abbreviations:** DM = diabetes mellitus, DME = diabetic macular edema, ETDRS = early treatment diabetic retinopathy study, CSME = clinically significant macular edema, CLP = conventional laser photocoagulation, VEGF = vascular endothelial growth factor, BCVA = best-corrected visual acuity, ANOVA = analysis of variance, VA = visual acuity

## Introduction

Diabetes mellitus (DM) has an increasing prevalence all over the world. The Global Burden of Disease Study 2017 states that the incidence and prevalence of DM were 22.9 million, and 476.0 million, respectively in 2017, with a projection to 26.6 million and 570.9 million in 2025 [**1**]. The overall prevalence of diabetic macular edema (DME) is about 7% and it means that at least 40 million people will probably suffer from DME in the near future [**2**].

DME is the most common cause of acquired visual loss in this population and hence, treatment of DME is of great importance. After Early Treatment Diabetic Retinopathy Study (ETDRS) defined clinically significant macular edema (CSME) and its treatment with laser photocoagulation [**3**,**4**], conventional laser photocoagulation (CLP) has become the only treatment option. Currently, anti-vascular endothelial growth factor (anti-VEGF) injections are the most preferred treatments in DME. Other new treatment modalities, such as intravitreal corticosteroid injections or implants, subthreshold micropulse lasers have also been introduced for DME treatment. Although these new treatments became increasingly widespread all over the world, they have some disadvantages compared with CLP. These treatments require several injections and/or treatment sessions and control examinations. They are expensive and necessitate good patient attendance. Moreover, some eyes with DME can be refractory to these treatments [**5**]. 

In this study, we aimed to investigate the long-term effectiveness and safety of CLP and to evaluate its potential to be a sustainable option in the treatment of DME.

## Materials and methods

In this retrospective study, medical records of diabetic patients referred to the retina department were reviewed. This study was conducted according to the Declaration of Helsinki and was approved by the Institutional Review Board (Approval No: 48670771-903.99). The patients who had non-proliferative diabetic retinopathy and DME and who were treatment-naive were included in the study. The patients with proliferative diabetic retinopathy or who progressed to the proliferative stage during treatments were excluded. Other exclusion criteria were history of cataract surgery for the last one year, history of another intraocular surgery, glaucoma or other ocular diseases including intraocular inflammations, eyes with nonperfusion areas within the perifoveal capillary network. Patients with hemoglobin A1c > 64 mmol/ mol (> 8%) at presentation were defined as having uncontrolled DM and were excluded from the study. The presence of diastolic blood pressure over 100 mmHg and/or chronic kidney failure were other reasons for exclusion.

Best-corrected visual acuity (BCVA) was measured by the Snellen visual acuity chart. Routine, complete ophthalmological examination, colored fundus photography and fundus fluorescein angiography were performed at baseline and follow-up examinations. Follow-ups took place 3 weeks after the 1st treatment, every 3 months for the first year, and every 6 months after the first year.

Eyes with focal or diffuse edema defined as CSME were treated with argon green (514 nm) or yellow dye (570 nm) laser randomly. CSME was defined accordingly to ETDRS protocol as retinal thickening at or within 500 μm of the center of the macula; hard exudates at or within 500 μm of the center of the macula, if associated with adjacent retinal thickening; or a zone or zones of retinal thickening one disc area in size, at least part of which was within one disc diameter of the center of the macula [**3**]. Diffuse macular edema was classified into cystoid and non-cystoid types. Successive treatments were performed with the same wavelength laser as the first treatment of the eye. Topical anesthesia was applied to all patients. Grid or modified grid laser photocoagulation was performed in the treatment of diffuse macular edema. In grid photocoagulation, 200 µm and 0.1-sec laser spots were used on the thickened retinal area by forming mild severity of laser burns and leaving one spot interval among laser spots. In focal treatment, laser spots of 100 µm and 0.1 sec were applied on every microaneurysm by obtaining slight whitening at the level of retinal pigment epithelium, subsequently, whitening, or darkening of the microaneurysm was observed with the application of additional 50 or 100 µm spots. In modified grid treatment, focal treatment was added to grid treatment. Persistent retinal thickening or new lesions detected during follow-up examinations were treated with additional laser photocoagulation.

*Statistical Analysis*

SPSS for Windows was used for statistical analysis. The effect of laser treatment on BCVA, correlations with variables related to patients and treatment were analyzed with this statistics program. Students t, Mann Whitney U, paired t, and ANOVA tests were used for comparisons. p < 0.05 was accepted for statistical significance.

## Results

The study comprised 133 eyes of 81 patients. One eye of 29 patients and both eyes of 52 patients were included. Forty (49.4%) patients were females, 41 (50.6%) patients were males. The mean duration of DM was 10.88 years (ranged from 6 months to 29 years). The mean follow-up was 28.26 months (ranged from 3 to 100 months). Yellow dye laser was applied to 17 eyes and argon green laser was applied to 116 eyes. While 112 eyes (84.2%) required treatment once, 17 eyes (12.8%) were treated twice and four eyes (3%) were treated three times (mean 1.19 ± 0.46). **Table 1** shows the number of eyes and applied laser treatment techniques.

**Tabel 1:** Applied laser treatment techniques and the number of the eyes

Treatment Technique	Number of Eyes
Only Focal	35
Only Grid	35
Only Modified Grid	42
Focal + Focal	2
Focal + Grid	3
Focal + Modified Grid	1
Grid + Grid	4
Grid + Modified Grid	4
Modified Grid + Modified Grid	3
Focal + Focal + Focal	1
Focal + Grid + Focal	1
Grid + Modified Grid + Modified Grid	2

BCVA change after treatment was analyzed and three eyes with BCVA lower than 0.10 were exempted from the analysis. Patients were divided into two groups according to baseline BCVA lower than or equal to 0.50 or more than 0.50. The first group with lower BCVA responded to the treatment better and BCVA after the laser treatment showed a statistically significant increase, whereas the second group with BCVA of more than 0.50 did not show a statistically significant change (**Table 2**).

**Tabel 2:** Change in BCVA according to baseline BCVA level

	Baseline BCVA < = 0.50	Baseline BCVA > 0.50
Baseline BCVA *(mean ± SD)	0.36 ± 0.01	0.78 ± 0.13
Last BCVA *(mean ± SD)	0.46 ± 0.21	0.74 ± 0.23
	p=0.001	p=0.070
*BCVA is expressed in decimal system BCVA = best-corrected visual acuity, SD = standard deviation		

**Fig. 1:**
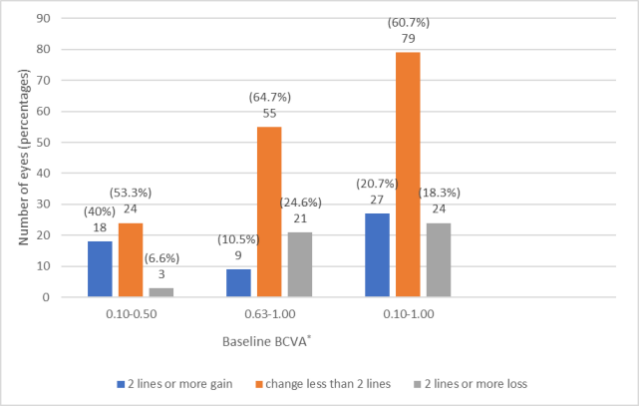
BCVA change at the last follow-up examination. In the first group with baseline best-corrected visual acuity (BCVA) between 0.10 and 0.50, conventional laser photocoagulation (CLP) stabilized BCVA within 2 lines in 24 eyes (53.3%), increased more than 2 lines in 18 eyes (40%), and decreased in three eyes (6.6%). In the second group with baseline BCVA between 0.63-1.00, CLP stabilized BCVA within 2 lines in 55 eyes (64.7%), increased more than 2 lines in nine eyes (10.5%) and decreased more than 2 lines in 21 eyes (24.6%). In total analysis, 27 eyes (20.7%) showed BCVA increase of 2 lines or more, 79 eyes (60.7%) stabilization within 2 lines, and 24 eyes (18.3%) loss of 2 lines or more, respectively. *BCVA = best-corrected visual acuity

When BCVA change of all eyes in the study was evaluated, the mean baseline BCVA was 0.63 ± 0.24 and the mean BCVA at the last visit was 0.65 ± 0.26 and the difference was not statistically significant (p > 0.05). At the last follow-up, CLP treatment preserved BCVA in 79 eyes (60.7%) and improved more than 2 lines in 27 eyes (20.7%) (**Fig. 1**).

BCVA change was analyzed according to the applied laser types. BCVA change in 113 eyes treated with argon green laser did not show a statistical significance (Baseline BCVA, 0.64 ± 0.23; last BCVA, 0.64 ± 0.26; p=0.94). Seventeen eyes treated with yellow dye laser showed statistically significant improvement in BCVA after treatment (Baseline BCVA, 6.23 ± 0.28; last BCVA, 0.70 ± 0.25; p=0.03).

Eyes with focal macular edema had the highest baseline BCVA, followed by diffuse non-cystoid macular edema and cystoid macular edema, respectively. However, BCVA change after laser treatment did not demonstrate a statistically significant difference in any type of DME (**Table 3**).

**Tabel 3:** BCVA change after laser photocoagulation treatment according to types of DME

	Focal DME (n=40)	Non-cystoid diffuse DME (n=65)	Cystoid diffuse DME (n=25)	F	p
Baseline BCVA * (mean ± SD)	0.70 ± 0.23	0.66 ± 0.22	0.46 ± 0.21	9.09	0.0002
Last BCVA * (mean ± SD)	0.70 ± 0.25	0.68 ± 0.24	0.48 ± 0.26	6.87	0.0015
	p=0.857	p=0.534	p=0.824		
*BCVA is expressed in decimal system BCVA = best-corrected visual acuity, DME = diabetic macular edema, SD = standard deviation					

BCVA changes were also evaluated according to type and duration of DM and received systemic treatments (**Table 4**). While the systemic treatment type and duration of diabetes did not show a statistically significant effect on BCVA change, BCVA in patients with DM type 1 revealed a statistically significant increase after laser photocoagulation treatment.

**Tabel 4:** BCVA changes according to systemic treatment type, duration, and type of DM

	Insulin (n=98)	OAD (n=32)	DM < = 10 y (n=71)	DM > 10 y (n=59)	Type I DM (n=7)	Type II DM (n=123)
Baseline BCVA * (mean ± SD)	0.64 ± 0.24	0.61 ± 0.22	0.62 ± 0.24	0.65 ± 0.23	0.60 ± 0.33	0.64 ± 0.23
Last BCVA * (mean ± SD)	0.66 ± 0.24	0.59 ± 0.31	0.64 ± 0.26	0.65 ± 0.26	0.72 ± 0.24	0.64 ± 0.26
	p=0.206	p=0.647	p=0.320	p=0.852	p=0.022	p=0.814
*BCVA is expressed in decimal system BCVA = best-corrected visual acuity, DM = diabetes mellitus, OAD = oral antidiabetic drugs, SD = standard deviation, y = years						

In one eye with BCVA of 0.80 before treatment, exudative plaque arose after 33 months of the treatment, and BCVA decreased to 0.5 m finger counting level. This patient had the diffuse type of macular edema and was treated by modified grid laser technique twice with an 18 months-interval. No other adverse effect was observed in the study group.

## Discussion

Currently, anti-VEGF intravitreal injections are the first-line treatment in DME. Steroid injections, subthreshold micropulse lasers have also been introduced as alternative treatments [**6**]. Albeit all these new treatments have started to predominate in clinical practice, CLP could not be replaced entirely in the treatment of DME and it is used as a single treatment or a part of combination therapy. In the current study, we reviewed the outcomes of CLP in the treatment of DME when none of the currently used other options for treatment were present. Our study showed a BCVA increase of 2 lines or more in about 21% of the eyes and stabilization within 2 lines in about 61% of the eyes. Laser photocoagulation demonstrated very good efficacy in the preservation of visual acuity (VA) in CSME including center-involved types in more than 80% of the eyes. These outcomes were in line with ETDRS, which reported that immediate laser photocoagulation in eyes with CSME could reduce the percentage of eyes with moderate visual loss by at least 50% and VA improved in 16%, remained unchanged in 77%, and worsened in 7% of the treated eyes [**3**]. In a study by Lee et al., laser photocoagulation improved VA in 14.5%, unchanged in 60.9% of the eyes with diffuse DME [**7**]. Similarly, Scott et al. evaluated the effect of focal/ grid photocoagulation on VA in eyes with non-center involved CSME and 75% of the patients preserved 20/25 or better visual acuity at one year after treatment [**8**].

Several studies reported that baseline BCVA was a strong predictor of BCVA changes after laser photocoagulation in DME and patients with poorer VA achieved greater gains than those with better baseline vision [**9**,**10**]. Comparably, improvement in BCVA was significant and better in the group with lower baseline BCVA in our study.

We compared VA change among groups classified according to types of edema; focal, non-cystoid diffuse edema, and cystoid diffuse edema and the results did not reveal any effect of edema type on VA change although baseline VA demonstrated a statistically significant difference between groups, the best with focal DME and the worst with cystoid diffuse DME. 

In the last decade, new laser models called subthreshold micropulse lasers have been introduced for the treatment of diffuse DME. Those new lasers aimed to deliver less thermal energy and claimed to create less photothermal damage to the neurosensory and inner retina than continuous-wave conventional lasers and hence, to induce less undesirable side effects such as visual field defects, epiretinal fibrosis, and choroidal neovascularization in the area of the laser scar. Although some studies reported better BCVA with subthreshold micropulse lasers, several studies comparing with conventional lasers stated a clinically insignificant difference in improving VA [**11**-**14**].

With the presentation of the first anti-VEGF pegaptanib for the treatment of DME, bevacizumab, ranibizumab, and aflibercept were introduced into the clinical practice sequentially. Several studies showed improvement in VA in center-involved DME [**15**-**17**]. However, Baker et al. compared the outcomes of initial management with aflibercept or with laser treatment or observation and given aflibercept only if visual acuity worsened in eyes with center-involved DME and good VA. They found no significant difference in vision loss at 2 years among three groups and thus no superiority of aflibercept injections over laser photocoagulation [**18**]. Anti-VEGF treatment necessitates several monthly injections to achieve desirable results during treatment of DME and causes treatment burden both financially and related to patient attendance [**16**]. Moreover, every injection may carry some risks such as endophthalmitis, cerebrovascular accidents even if they are seen very rarely [**19**,**20**].

## Conclusion

In conclusion, our study demonstrated that CLP stabilized or improved visual acuity in 81% of the eyes for a long-term follow-up. Although anti-VEGF injections are the main treatment modality in center-involved DME and additional treatments such as corticosteroid injections and subthreshold micropulse lasers have been presented as alternative treatments, CLP still has a role in the treatment of DME because of its good outcomes, low cost and fewer treatment sessions for patients who have difficulties in attending appointments. The limitation of our study is its retrospective design and lack of control groups.

**Conflict of Interest statement**

The authors state no conflict of interest.

**Informed Consent and Human and Animal Rights statement**

Informed consent has been obtained from all individuals included in this study.

**Authorization for the use of human subjects**

Ethical approval: The research related to human use complies with all the relevant national regulations, institutional policies, is in accordance with the tenets of the Helsinki Declaration, and has been approved by the institutional review board of the University of Health Sciences, Sultan 2. Abdulhamid Han Training and Research Hospital, Istanbul, Turkey (Approval No: 48670771-903.99).

**Acknowledgments**

None.

**Sources of Funding**

This study received no public or private support.

**Disclosures**

None.
